# A Systematic Review on Synthetic Medical Images Generation—Recent Trends and Future Opportunities

**DOI:** 10.3390/diagnostics16142166

**Published:** 2026-07-10

**Authors:** Yumna Waheed, Muhammad Nouman Noor, Imran Ashraf

**Affiliations:** 1 Department of Artificial Intelligence and Data Science, National University of Computer and Emerging Sciences, Islamabad 44000, Pakistannouman.noor@isb.nu.edu.pk (M.N.N.); 2 Computer Engineering Lab, Quantum and Computer Engineering Department, Faculty of Electrical Engineering, Mathematics and Computer Science (EEMCS), TU Delft, 2628 CD Delft, The Netherlands

**Keywords:** medical image generation, GAN, diffusion models, variational autoencoders, loss functions, synthesis

## Abstract

**Background/Objectives:** Generative Models have revolutionized the synthesis of complex, realistic medical images. However, obtaining annotated, high-quality datasets is challenging and expensive due to privacy concerns, high human annotation costs, and data scarcity. This systematic literature review (SLR) provides a comparative overview of Generative AI models used for medical image generation, categorizing the research into GANs, Diffusion Models, and Autoencoders used for medical image generation and GAN loss functions. **Methods:** This systematic review followed the PRISMA 2020 guidelines. Studies published between 2021 and 2026 were retrieved from selected scientific databases and screened using predefined inclusion criteria. We selected 120 papers published in reputable databases and journals. The review examined generative modeling approaches aimed at addressing data scarcity and annotation limitations in medical imaging, including GAN-based adversarial synthesis, diffusion-based iterative denoising, and Autoencoder-based latent representation learning, along with an in-depth analysis of GAN loss functions. **Results:** GANs were the most widely used approach (36% of the reviewed studies), achieving the lowest FID scores (15.552–31.349). Diffusion Models (26% of the reviewed studies) showed superior structural fidelity, achieving SSIM values of up to 0.915, whereas GANs achieved 0.593 on brain MRI datasets. In addition, hybrid loss functions combining perceptual, structural, and pixel-level terms resulted in improved image quality performance (PSNR = 35.6; SSIM = 0.95). **Conclusions:** The analysis showed that GAN-based generative models produce visually realistic images with detailed textures, whereas Diffusion Models generate high-resolution images with superior structural fidelity but require substantial computational resources for training and image generation. Furthermore, the clinical implications, limitations, and challenges of Generative AI models were discussed. Despite these advances, the medical imaging field still faces several challenges, including the need for annotated medical datasets, high-quality realistic images for training, and limited data availability.

## 1. Introduction

In the past few years, Generative AI models have been demonstrated in clinical imaging. In healthcare, the generation and enhancement of medical images play a significant role in image diagnosis and classification. Therefore, the ability to generate realistic high-resolution medical images is a crucial step in accurate treatment planning. Generative modeling, as a form of unsupervised learning, is a field that researchers have applied to various imaging generation tasks. Recent contributions, including Variational Autoencoders (VAEs) [1], Generative Adversarial Networks (GANs) [2], and Diffusion Models [3], are based on probabilistic modeling, adversarial training, and iterative denoising processes. These models learn complex data distributions to generate realistic and high-quality medical images. Despite these advancements, medical imaging faces several challenges, such as low contrast, small lesion visibility, noisy images, structural variability, mode collapse, and data scarcity, reducing the ability to generate high-resolution images [4]. For example, wireless capsule endoscopy, an imaging modality, captures thousands of frames from the gastrointestinal tract [5]. The limited perspective and image quality lead to missing structural and anatomical information for analysis and generation, making it difficult to obtain high-quality images [6].

Generative models have been recognized as a pivotal innovation in AI research, offering unique capabilities that differentiate them from traditional approaches [7]. Medical image synthesis requires advanced techniques for realistic synthesis, reconstruction, and enhancement. These generative models have demonstrated significant potential in the field of medical image generation by learning complex patterns and data distribution. Diffusion Models have been used to produce quality images, preserving the global structure and semantics of the image [8,9,10]. GANs have been employed to produce 2D or 3D images, improving image sharpness and reducing artifacts in medical data [11,12].

Although publicly available annotated medical datasets such as Kvasir, BraTS, ChestX-ray, and Hyper-Kvasir have advanced medical image diagnosis, they exhibit structural constraints and lack diversity [13]. Improving the quality and applicability of medical image generation requires a structured understanding of how model architectures mitigate these challenges. Existing systematic reviews are fragmented in their evaluation of model optimization and provide limited evidence on how GAN loss formulations, mode collapse, vanishing gradients, architectural hyperparameters, and optimization inconsistencies collectively affect the reliability of synthetic medical image generation [14,15,16,17,18,19,20,21,22]. Although prior literature focuses on model architectures and datasets, providing a limited overview of various GAN loss functions, Wasserstein distances, least-squares formulations, hinge losses, and hybrid objective functions within a unified framework. The limited use of GAN loss functions across diverse datasets and the widespread absence of standardized reporting for training convergence profiles further obscure the true reproducibility of published synthetic frameworks. Furthermore, no prior review has systematically evaluated how different GAN loss functions affect image quality, training stability, and convergence in the context of medical imaging.

To examine GANs, Diffusion Models, and VAEs in image generation, a systematic literature review is crucial. In this SLR, we review the generative models for medical image generation and their variants, the impact of the loss function in GAN, and the challenges in the existing literature. We also analyze various generative architectures and medical image modalities by conducting a survey on medical image generation models. Despite the significant success of generative models in image generation, which allows the model to produce high-quality results, generative models suffer from high computational cost, mode collapse, and unstable training. In this review, we also discuss the problems in GANs and associated loss functions. In this SLR, we present the following key contributions:

- We provide a comprehensive analysis of Generative AI models, including Variational Autoencoders, GANs, and Diffusion Models in medical imaging.
- An in-depth evaluation of GAN loss functions in image generation to improve quality, stability, and convergence.
- We discuss GAN architectures commonly used in medical imaging and their applications across various medical imaging modalities.
- We systematically examine key training and data challenges identified based on the synthesis of the 120 included studies. Specifically, mode collapse, vanishing gradients, and training instability in GAN-based models are analyzed in Section 5.3.1, Section 5.3.2, and Section 5.3.3 respectively. Section 5.2 provides a relative comparison of computational cost over model categories. Data scarcity and imbalanced datasets are identified as recurring limitations across the included studies and are discussed in the context of Section 5. The challenges reported throughout this review were identified through systematic thematic synthesis of the limitations sections of all 120 included studies. These challenges were identified based on the systematic extraction and synthesis of reported limitations using the data extraction approach detailed in Section 2.5.

This paper is organized as follows. Section 2 outlines the methodology, Section 3 presents various generative models and hybrid approaches, Section 4 presents GAN loss functions applied in image generation and the strengths and weaknesses of commonly used loss functions, and Section 5 outlines the discussion. Section 6 outlines limitations and Section 7 concludes the review.

## 2. Methods

The methodology of this systematic literature review (SLR) was conducted in accordance with the PRISMA 2020 guidelines [23] and the completed PRISMA 2020 Checklist is provided in the Supplementary Materials. The methodology presents the technique and criteria to assess articles. It is divided into research questions, search strategy, search outcomes, quality assessment criteria, dataset overview, and data extraction strategy. The protocol for this review was not recorded in any other publicly accessible registry (such as PROSPERO); all methodological procedures were conducted in accordance with PRISMA guidelines.

### 2.1. Data Collection

**Figure 1:**
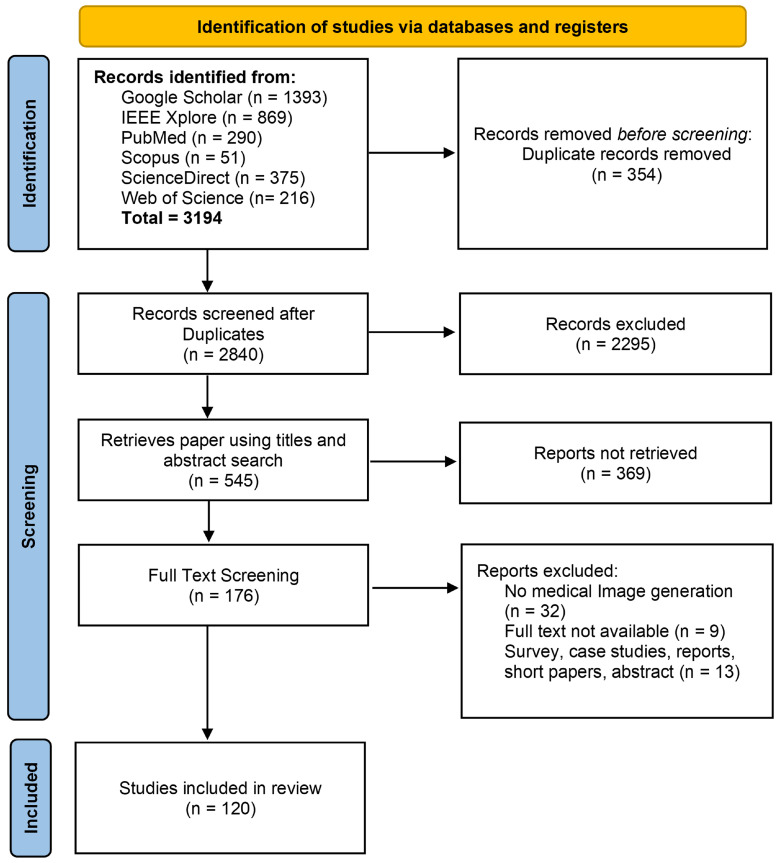
PRISMA diagram.

The systematic search identified papers from selected databases such as IEEEXplore, Google Scholar, ScienceDirect, PubMed, Web of Science, and Scopus, and extracted a total of 3194 papers. After the removal of duplicates, a total of 2840 papers were screened. A total of 545 papers were retrieved based on title and abstract screening, then reduced to 176 papers via full-text review. In the final stage, 120 papers were included after applying all inclusion and exclusion criteria. PRISMA diagram outlining identification, screening, eligibility, and inclusion is presented in Figure 1.

### 2.2. Research Question

This SLR answers the following questions:

**RQ1:** What are the generative model techniques employed for the synthesis of medical images in different modalities?
**RQ2:** Which loss functions are commonly used in GANs for improving the quality and stability of synthetic image generation?
**RQ3:** Which generative models have been applied in clinical settings?
**RQ4:** How are generative models used to overcome data limitations?

### 2.3. Formulate Search Query

The papers were identified from six well-known databases using a systematic search. Each database was accessed directly through official web interfaces. To identify relevant studies, we applied the following search queries: “synthetic image generation”, “GANs in medical imaging”, and “data augmentation”. We also used combined keywords to enhance the following search query:

- (“Autoencoder” OR “VAE” OR “Variational Autoencoder” OR “Variational Autoencoders” OR “GAN” OR “GANs” OR “Generative Adversarial Network” OR “Generative Adversarial Networks” OR “Diffusion Model” OR “Diffusion Models” OR “Latent Diffusion Model” OR “DDPM” OR “score-based generative model” OR “score matching”) AND (“medical image generation” OR “medical image synthesis” OR “clinical image synthesis” OR “synthetic medical images” OR “modality translation” OR “cross-modality synthesis”) [Filter: 2021–2026, English]
- (“GAN” OR “GANs” OR “Generative Adversarial Network”) AND (“loss function” OR “objective function” OR “adversarial loss” OR “adversarial training loss” OR “perceptual loss” OR “Wasserstein loss” OR “hinge loss” OR “cycle-consistency loss”) AND (“medical image synthesis” OR “medical image generation” OR “clinical image generation” OR “synthetic imaging”) [Filter: 2021–2026, English]
- (“score-based generative model” OR “score matching” OR “text-to-image” OR “Stable Diffusion” OR “privacy-preserving” OR “modality translation” OR “cross-modality synthesis” OR “domain adaptation”) AND (“medical image generation” OR “medical image synthesis” OR “clinical imaging” OR “radiology image synthesis”) [Filter: 2021–2026, English]

Database-specific search settings were applied to limit searches where applicable. The search strategy was designed using a PICOC-guided approach, focusing on generative AI-based medical image generation. Table 1 illustrates the overview of the literature selected using the PICOC framework.

### 2.4. Eligibility Criteria

In this systematic literature review, we defined inclusion and exclusion criteria to ensure the selection of relevant and high-quality papers. Table 2 illustrates the eligibility criteria:

Applying these criteria, the selection followed a PRISMA 2020 multi-stage workflow.

- Title and Abstract Screening: Initial screening of studies that were not related to medical imaging to remove irrelevant literature.
- Full-Text Review: The remaining studies were assessed for data collection details, methodology clarity, and evaluation results.
- Backward and Forward Search: With PRISMA guidelines, a supplementary backward and forward reference search was conducted to ensure that all relevant studies were included.

Table 3 presents an overview of the generative model architectures, including GAN-based approaches, Diffusion-based methods, and Autoencoder-based architectures used in this review.

**Figure 2:**
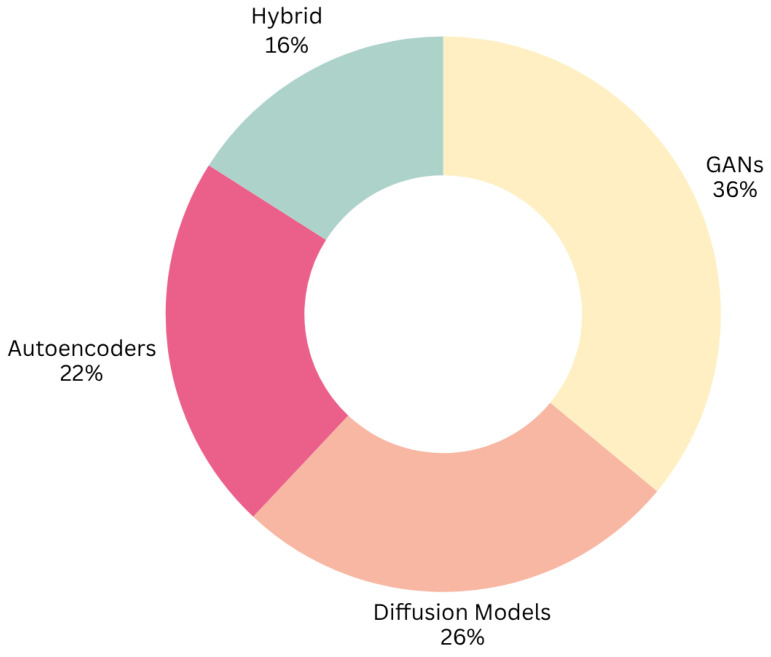
Distribution of Generative Model Architectures.

The pie chart in Figure 2 highlights the proportion of studies that employed different generative models in the reviewed literature.

### 2.5. Data Extraction

A data extraction form was designed to maintain reliability across all 120 included studies. All relevant data fields were systematically and manually extracted from each included study by the primary reviewer (Y.W.). The extracted data were subsequently reviewed and independently validated by the second reviewer (M.N.N.) in a supervisory role and validation on a random subset of the included studies (20%, *n* = 16), and by the third reviewer (I.A.) as part of formal analysis and validation, to ensure consistency, completeness, and reproducibility. Disagreements between reviewers were resolved through structured discussion or by consulting a third reviewer. The following data fields were extracted for systematically capturing methodological practices:

- Generative model type: GAN-based, Diffusion Model-based, Autoencoder-based, or Hybrid architecture.
- Medical imaging modality: MRI, CT, X-ray, endoscopy, ultrasound, PET, OCT, dermoscopy, or multimodal imaging.
- Datasets used: Public benchmark datasets (e.g., BraTS, Kvasir-v2, NLST, MedicalMNIST) or private datasets, including image count and domain specificity.
- Loss functions applied: Adversarial, perceptual, structural similarity, pixel-level, textural, content, or hybrid loss formulations used during GAN training.
- Data augmentation strategies: Geometric transformations, Gaussian noise, motion blur, or other augmentation techniques employed to expand training datasets.
- Performance metrics: Image quality metrics used in medical image generation: Fréchet Inception Distance (FID), Structural Similarity Index Measure (SSIM), Peak Signal-to-Noise Ratio (PSNR).

The challenges reported in this systematic review, such as mode collapse, vanishing gradients, training instability, data scarcity, dataset imbalance, and computational cost, were identified through systematic thematic synthesis of the limitations sections of all 120 included studies and discussed in Section 5.3.1, Section 5.3.2 and Section 5.3.3 and Section 2.6.

Due to heterogeneity in generative model architectures, imaging modalities, datasets, and evaluation metrics across the included literature, a quantitative meta-analysis was not feasible. Instead, findings were grouped by generative model category (GAN, Diffusion Model, Autoencoder, and Hybrid). The synthesized results are available in the Supplementary Materials.

### 2.6. Datasets Overview

**Figure 3:**
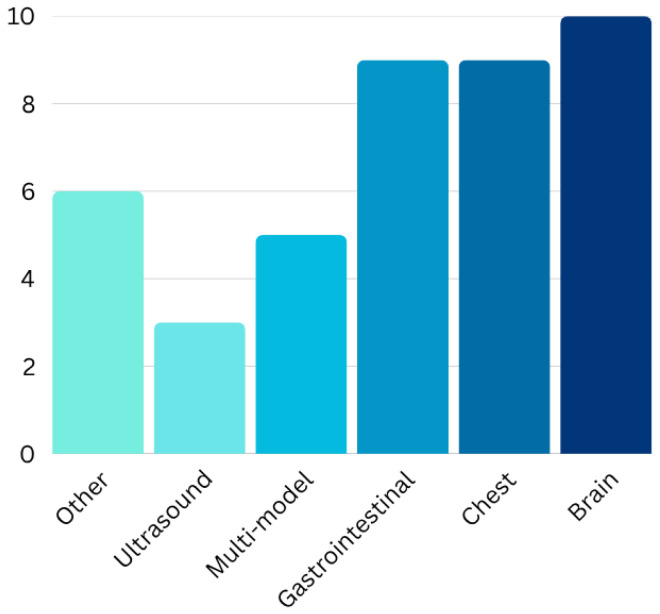
Distribution of datasets used in selected studies by imaging modality.

A number of studies have utilized small datasets, which impacts the ability to learn meaningful representations and complex data distributions in medical image synthesis. The medical images used in many studies are domain-specific. For model training, publicly available datasets were employed in many studies, and some studies used multimodal datasets. The summary of datasets used in this review is shown in Table 4:

The most common models for medical image synthesis are Diffusion Models, Generative Adversarial Networks (GANs), and Variational Autoencoders (VAE). Studies have also applied data augmentation techniques such as geometric and color transformations, Gaussian noise, and motion blur to expand the training dataset. The bar chart in Figure 3 presents a comparison of various datasets from the selected studies and shows multiple types of imaging data.

**Figure 4:**
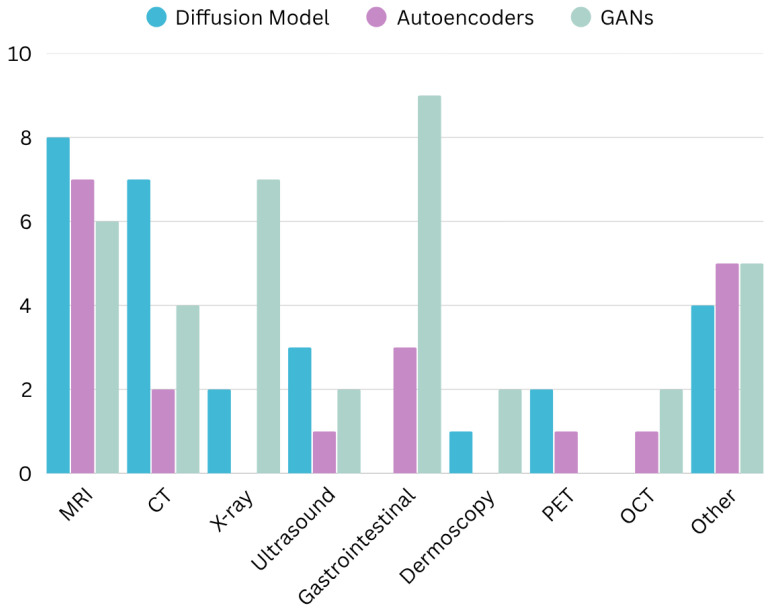
Selected studies across modalities and approaches.

Figure 4 illustrates the distribution of Diffusion Models, Autoencoders, and GANs across different medical domains. The bar chart shows the Gastrointestinal dataset as the leading area in this literature.

### 2.7. Quality Assessment and Risk of Bias

To ensure consistency in the evaluation of the selected studies, we applied a structured quality assessment (QA) framework, as illustrated in Table 5. This framework assesses each study on five predefined criteria: (1) clarity of the proposed generative model objectives, (2) completeness of the methodological framework, (3) relevance to medical image generation, (4) quality of evaluation metrics (e.g., FID, SSIM, PSNR), and (5) clarity of reported limitations and conclusions. The risk of bias and methodological quality of the included studies were evaluated by two reviewers (Y.W. and M.N.N.) using the customized 5-point quality assessment (QA) framework, as illustrated in Table 5.

**Figure 5:**
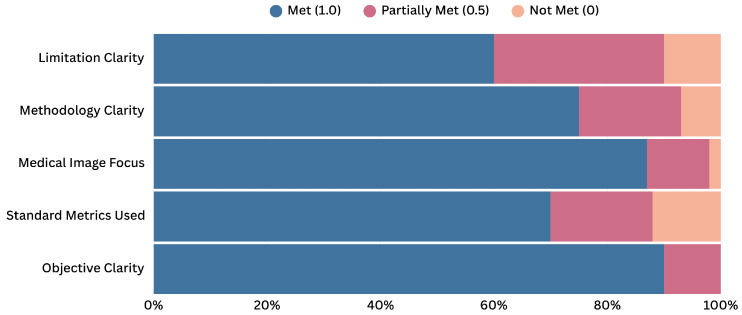
Risk of Bias Assessment across Selected Studies.

Each study was examined to determine whether it incorporated the key elements defined in the data extraction plan. Articles were selected based on the assigned scores of 1 (met), 0.5 (partially met), and 0 (not met) for the specified assessment criteria. As presented in Figure 5, the studies presented low risk of bias, with the majority of studies scoring 1.0 (Met) across these criteria. ’Standard Metrics Used’ and ’Limitation Clarity’ exhibited higher proportions.

## 3. Medical Image Synthesis with Generative Models

Numerous researchers have employed generative models to perform various tasks, including generation, reconstruction, segmentation, and denoising. This section discusses some generative models such as Generative Adversarial Networks (GANs), Diffusion Models, and Autoencoders, their applications, and imaging modalities in detail. A complete summary of study characteristics, including methods, datasets, model types, and evaluation metrics for all included studies, is provided in Supplementary Table S1.

### 3.1. Generative Adversarial Networks in Medical Image Synthesis

GANs were first introduced in 2014 [2] and have been used to learn data distributions for image generation through adversarial training. These methods use a generator and a discriminator to produce synthetic data that resembles real data. The model generates complex, high-dimensional data by transforming random noise into realistic samples. Various GAN architectures have been proposed, for instance, CycleGANs [36], which improve image translation by learning a mapping between two different image domains, such as in medical MRI-to-CT and CT-to-MRI, etc. By incorporating the cycle-consistency loss function, it ensures more accurate translation. StyleGAN can generate high-resolution synthetic medical images [37].

**Figure 6:**
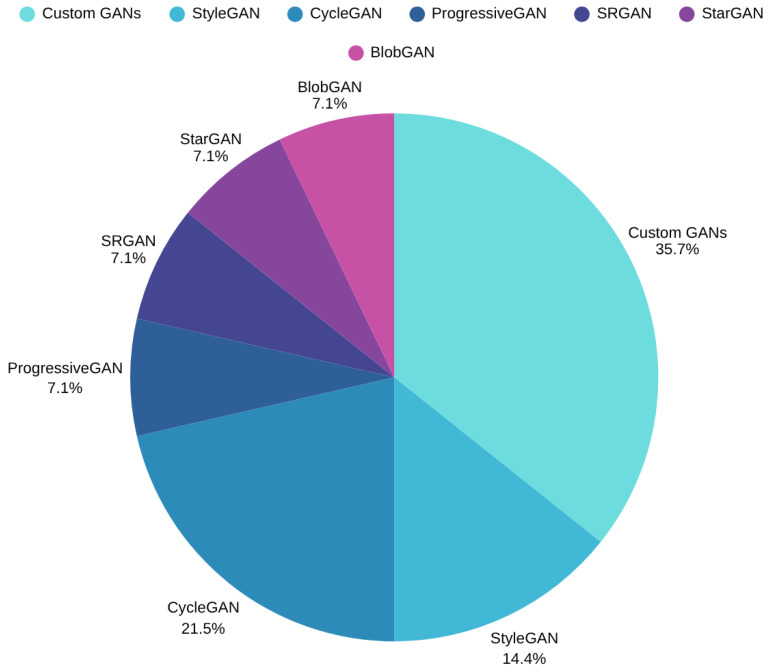
Distribution of GAN architectures.

Numerous researchers have applied GAN frameworks to medical image generation. For instance, to improve image generation, various advanced techniques have been incorporated into GANs. Maguluri et al. [38] proposed a PGAN that synthesizes chest images using residual connections and progressive upsampling. Performance metrics included SSIM and FID, which achieved 22.4 and 0.9, respectively. Zhang et al. [24] aimed to generate quality data using a GenSeg framework. The framework used multi-level optimization to achieve better performance. Feng et al. [39] leveraged a knowledge distillation GAN to address the data scarcity for medical image generation. The KGAN achieved SSIM of 0.593 and SCD of 1.229. Khatun et al. [30] aimed to generate medical images using quantum learning, named QIGL. Liu et al. [40] aimed to propose a RadImageGAN, a radiologic image generation across multiple modalities. They combined with BigDatasetGAN to produce multiclass, high-resolution synthetic medical images. Shah et al. [41] leveraged SRGAN, a super-resolution GAN, to improve image quality. To improve image resolution, perceptual and adversarial losses are used to preserve the texture and sharpness of medical images. Figure 6 illustrates the GAN architectures used in medical image generation.

**Figure 7:**
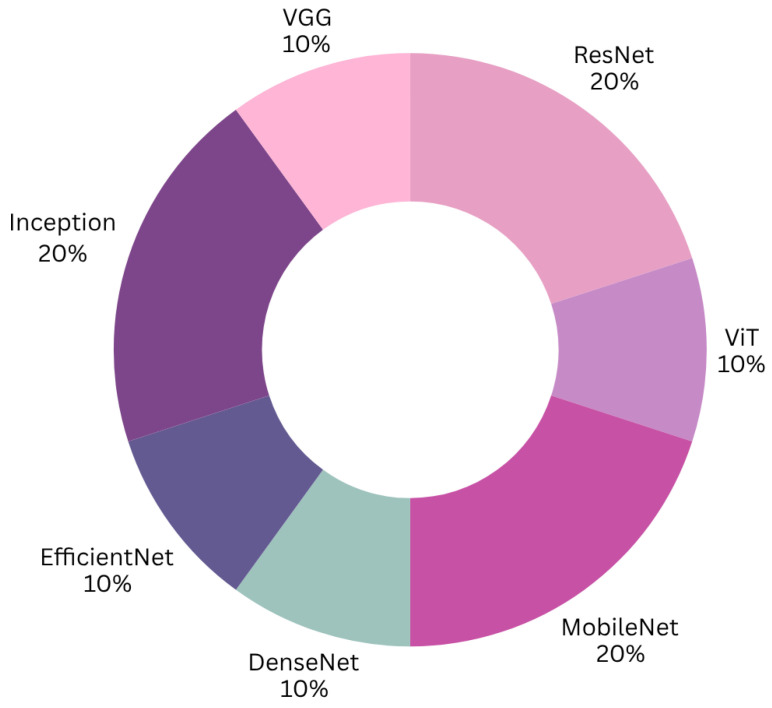
Distribution of CNN Architecture.

In [12], the authors Janutenas and Sesok applied the Viewpoint Attention Module and Perspective Transformation Module within StarGAN to augment endoscopic images. The goal was to improve multiclass classification accuracy against InceptionNet-V3, DenseNet-121, VGG-16, ResNet-50, and EfficientNetB7. Similarly, Magalhães et al. [11] applied a Deep convolutional GAN for endoscopic image augmentation. Their proposed approach was evaluated using ResNet50 and Vision Transformer (ViT) architectures for classification. A multiscale generative network was designed by Liu et al. [26] to generate super-resolution images. They combined five loss functions to improve endoscopic image quality and achieved 0.83 SSIM and 31.86 PSNR on the Kvasir dataset. Ayubi et al. [25] employed BlobGAN with self-attention for GI image generation. A self-attention block improves feature representation and image realism, and with an FID of 15.5, it enhances the quality of gastrointestinal images and improves classification systems. In another study, Prabu et al. [42] introduced a method to improve the classification of endoscopic images by utilizing a machine learning (ML) algorithm and a GAN, named EIC-MLA. The proposed method enhances image quality and GI disease classification, unlike traditional methods that achieve lower classification accuracy. Mao et al. [43] employed CycleGAN, a GAN-based framework, to transform low-normal-light and normal-low-light gastroscopic images. The study improved image brightness for clinical diagnosis using an illumination-aware attention module. Numerous studies have utilized CNN architectures to capture complex patterns and hierarchical features, improving classification, diagnosis, and image generation tasks. Xiao et al. [5] aimed to design a capsule endoscopy generation method using a special common attention GAN (SCAGAN). This method was used to capture external and internal features. To improve GAN performance, a DiffSeg method was employed. The experiment showed that SCAGAN enhanced model quality and achieved an FID of 31.349. Yoon et al. [44] employed a GAN-based system to learn SSL-specific features and generate realistic endoscopic images. The proposed method based on a CADe configuration with augmentation methods achieved an FID of 42.19 and a sensitivity of 93.98%. Lee et al. [45] improved detection of gastric cancer by integrating YOLOv8 with synthetic image generation using StyleGAN3. It achieves this by generating high-resolution gastric cancer images. Figure 7 shows the usage of different CNN models in the selected review:

In brain MRI synthesis approaches, researchers have utilized GAN-based methods, possibly due to their ability to generate sharp, highly realistic anatomical details. Mukherjee et al. proposed an aggregated GAN-based framework, denoted as AGGrGAN, designed to synthesize brain tumor MRI generation. Specifically, two variants of DCGAN [46] and a Wasserstein GAN (WGAN) [47] were incorporated into a unified framework to capture diverse feature representations from the input data. Furthermore, a style transfer mechanism was integrated into the generation pipeline to enhance the visual realism and structural resemblance of the synthesized images [48]. Onakpojeruo et al. proposed a novel Pix2Pix GAN-based framework for synthetic brain MRI generation, leveraging conditional image-to-image translation. The framework generates realistic brain tumor images through a conditional adversarial learning process, providing augmented training data for a Conditional Deep Convolutional Neural Network for multiclass brain tumor classification [49]. Hamghalam et al. proposed a conditional Generative Adversarial Network (cGAN)-based framework for brain tumor segmentation, employing two complementary Enhancement and Segmentation GAN (ESGAN) and Enhancement GAN (EnhGAN) models. To enhance the contrast of tumor sub-regions within the image, the authors utilized a contrast-enhancement process that adaptively calibrates voxel intensities within input patches [50]. Zhou et al. introduced a vector quantization GAN (VQ-GAN), designed to generate 3D brain tumor ROIs [51]. Wang et al. proposed a Selective State-Space Module, denoted as SelSSM, designed to synthesize multimodal MRI images across diverse GAN architectures. To improve anatomical consistency, the authors utilized a gradient-constrained adaptation that improves spatial alignment. The experiments demonstrated that SelSSM improves both image synthesis quality and segmentation performance [52]. Saimon et al. employed a conditional GAN (cGAN)-based framework, designed to generate synthetic brain tumor images. The study utilized these generated images for data augmentation by training multiple deep learning models on synthetic data to address class imbalance in brain tumor classification [53]. Lai et al. proposed a StyleGAN2-ADA-based framework, named StyleGAN2-ADA, designed to generate high-quality synthetic brain MRI images for data augmentation and privacy-preserving medical image sharing. Specifically, training set size variations were systematically incorporated into the model training process to analyze the impact on image fidelity and diversity metrics [54].

### 3.2. Diffusion Model in Medical Image Synthesis

Diffusion-based models are designed to generate realistic, high-quality, and diverse data by learning to reverse a noise-corruption process [3]. Many diffusion-based methods have been proposed to enhance reconstruction, segmentation, generation, and denoising from complex data distributions. Wang et al. [55] primarily discussed two models for medical image restoration. In this study, the authors categorized Diffusion Models into score-based models and denoising diffusion probabilistic models and utilized these to generate CT, MRI, endoscopic, and PET data. In the conditional DDPM method, Wang et al. [35] generated high-quality CT images using masks and labels. Better recovery of anatomical details was achieved using a super-resolution GAN, achieving a PSNR of 19.51.

**Figure 8:**
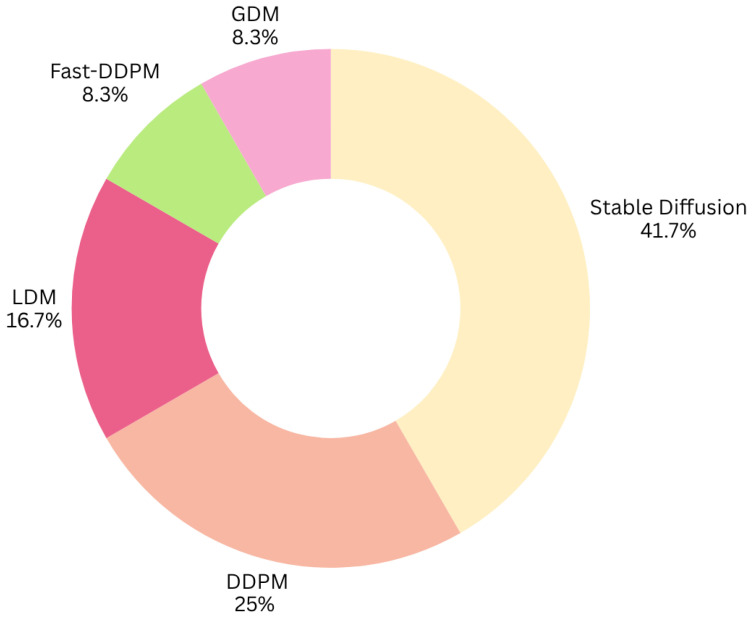
Diffusion Model Architectures Distribution.

Krishna et al. [31] proposed a denoising diffusion probabilistic model with multi-condition guidance for lung CT and mammography image synthesis. Performance metrics included AUC and F1 Score, achieving an AUC of 0.599 and an F1 Score of 0.319 on 10,000 synthetic images. In another study [8], Ma et al. introduced MDDPM, which used a multi-level UNet and a cross-attention mechanism to generate super-resolution MRI images. Approximate SSIM for Alzheimer MRI, Knee MRI, and Brain MRI datasets were 0.888, 0.899, and 0.915, respectively. Pan et al. [34] employed DDPM for the generation of cervical MRI images, named CSM-DPM. The method enhances the DDPM by incorporating Gaussian and point noise. Conditional diffusion-based mammogram synthesis, introduced by Montoya et al. [10], allows the controlled generation of images. Instead of relying on multiple separate models, which increase computational complexity, the proposed framework fine-tunes a pretrained Stable Diffusion Model to learn domain-specific and structural features, while Hung et al. [56] applied a conditional Denoising Diffusion Probabilistic Model (DDPM) for MRI super-resolution, inpainting, and CT/X-ray image synthesis. Liu et al. [57] presented a diffusion-based framework for fully automatic polyp image generation, named Polyp-Gen. This method employed a spatial-aware diffusion training to improve polyp sharpness and structure. Freiche et al. [58] fine-tuned Stable Diffusion to generate high-quality ultrasound images. Similarly, Kaleta et al. [59] employed stable diffusion for endoscopic image synthesis. This method is based on image-to-image translation and achieved an IOU of 69.76% in another study. Figure 8 summarizes the usage of different diffusion model architectures in the reviewed studies.

Onakpojeruo et al. [60] introduced a denoising diffusion model (DDM) based framework designed for synthetic data generation in brain tumor classification. Leveraging a denoising Diffusion Model (DDM) and a deep convolutional neural network for synthetic image generation and brain tumor classification, the method aims to enhance the data diversity and improve classification performance. Moshe et al. [61] presented a diffusion-based framework for brain tumor MRI synthesis and classification generalization, employing Denoising Diffusion Probabilistic Models (DDPM) and mutual information (MI) regularization to generate realistic and diverse synthetic MR images. Kebali et al. [62] proposed a slice-based latent diffusion framework for 3D multimodal medical image and mask synthesis. The proposed approach enables simultaneous generation of volumetric images and corresponding multi-label tumor masks through a diffusion-driven generative process, providing enhanced structural consistency and diversity for downstream tumor segmentation tasks. Similarly, Mirowski et al. [63] introduced a diffusion-based framework for brain lesion segmentation, which leverages ControlNet [64] and a custom-designed Diffusion Model to synthesize realistic MRI brain images. In the proposed framework, a diffusion-based generative network is employed to synthesize realistic MRI brain images for lesion segmentation, and a U-Net segmentation network is utilized to learn robust feature representations.

Several methods have been proposed to reduce computational cost and training time. For instance, Jiang et al. [33] introduced Fast-DDPM with the aim of reducing training for realistic image-to-image generation. The author designed a noise scheduler by incorporating uniform and non-uniform sampling. The results show that the model achieved a high PSNR and SSIM across different tasks. Similarly, Zhang et al. [65] proposed a GDM, a geodesic path to generate medical images. The model in this paper reduced 50× and 10× training times compared with DDPM and Fast-DDPM, respectively.

### 3.3. Autoencoder in Medical Image Synthesis

**Figure 9:**
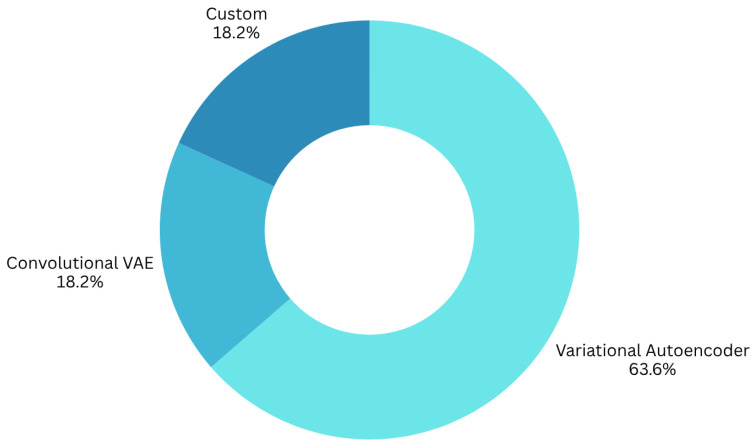
Distribution of Autoencoder types.

Numerous researchers have employed Autoencoders to reconstruct and generate medical images. Diamantis et al. [27] proposed an EndoVAE method, which utilized Variational Autoencoders to generate endoscopic images. The model was evaluated on the KID dataset and generated images with improved diversity and quality, enhancing the performance of the endoscopic classifier. Figure 9 provides an overview of the distribution of various Autoencoders used in the selected studies.

Fuente et al. [66] introduced a class-specific image generation Variational Autoencoder method with the aim of generating realistic images for classification using Variational Autoencoders. The proposed method increased accuracy by 18% and improved performance on imbalanced datasets. Diamantis et al. [28] proposed TIDE, a unique VAE that generates diverse and high-quality images, incorporating residual connections. TIDE was evaluated on the KID and Kvasir-Capsule datasets and achieved AUCs of 0.894 and 0.802, respectively. Jaen et al. [67] combined two techniques: style transfer and convolutional VAE to produce cardiac MR images. The combined technique enhances image quality by reducing blurring effects. Hangaragi et al. [68] presented a method to generate lung cancer images by using CAE. The model learns a latent representation for generating realistic chest images, improving classification and augmentation. Huang et al. [69] introduced a L-VAE method, a unique Variational Autoencoder for lumbar spine generation. Rais et al. [32] introduced CAT-VAE, a combination of a Cross-Attention Transformer and a Variational Autoencoder to enhance image quality and capture both local and global details. Their findings showed that CAT-VAE achieved an accuracy of 97.50%, which outperformed in classification tasks. Similarly, Dorent et al. [70] proposed MHVAE, a unique multimodal hierarchical VAE for MR/iUS synthesis, outperforming existing models in SSIM, PSNR, and LPIPS. Gan and Wang et al. [71] introduced adversarial learned VAE for esophageal OCT synthesis. It employed GAN and VAE to capture tissue details and reduce noise, resulting in higher image quality than existing models. Choudhury et al. [72] introduced a new method for TTE to CMR image synthesis. The method utilizes an Autoencoder with a vision transformer to generate CMR from TTE by learning patterns from Transthoracic Echocardiography (TTE) data.

### 3.4. Hybrid Generative Model in Medical Image Synthesis

There are several studies in the medical imaging domain that explore the use of Variational Autoencoders and GANs for image synthesis. Liang et al. [73] achieved improved quantitative results in thyroid ultrasound image generation using an alpha-WGAN-GP framework. Furthermore, the framework also trains a UNet to achieve better segmentation results. Yang et al. [74] presented a hybrid GAN-guided conditional diffusion for enhanced CT images. GANs-CDM use image-based conditioning to guide the Diffusion Model for refined image synthesis. Experimental results show that images generated by GANs-CDM can simulate realistic contrast-enhanced CT images and anatomical structure for conditional medical image synthesis. In [75,76,77], the authors combined Variational Autoencoders and GANs for image synthesis. Rguibi et al. [75] improved feature learning and image generation quality by integrating GANs with VAE. In another study, Sa et al. [77] used a VAE-GAN for knee MRI image synthesis using hybrid generative models, improving FID and MSE for image synthesis. Feihong et al. [76] used VAE in the GANs generator for ASL image synthesis. A detailed experiment of dementia disease diagnosis is provided, along with a comprehensive comparison of multiple models. Cao et al. [78] designed a multimodal framework for generating modality-specific medical images, namely AE-GAN, achieving high PSNR and SSIM in both generation and segmentation tasks. A detailed experiment of various models on three different datasets is provided, along with an analysis of the effect of the hybrid loss function. Afnan et al. [79] integrated UNet, VAE, and CycleGAN for brain CT and MRI images using bidirectional learning, namely VAE-CycleUNet. Performance metrics including MAE, PSNR, and SSIM were employed, assessing the model on brain MRI and CT dataset. In a related study, Fatima et al. [80] combined GAN with Autoencoder for lung ultrasound image synthesis using a conditional latent space to guide GAN training.

### 3.5. Emerging Trends in Generative Medical Imaging

Numerous researchers have explored emerging trends in generative medical imaging and discussed three prominent directions, including Latent Diffusion Models (LDMs) in healthcare, multimodal Generative AI for text-to-medical image synthesis, and privacy-preserving generative frameworks.

#### 3.5.1. Latent Diffusion Models in Healthcare

Latent Diffusion Models (LDM) [81] are applied in healthcare for image synthesis. Unlike conventional Diffusion Models, which operate directly on high-resolution image pixels, LDMs perform the forward and reverse diffusion process within a compressed latent space. In another study, Wang et al. [82] presented a unified text-to-medical image generation framework (MINIM). A latent Stable Diffusion Model was used to generate OCT, fundus, chest CT, and chest X-ray images. Performance metrics including FID, MS-SSIM, and IS were employed, achieving an FID of 65.3 for OCT. Similarly, Martyniak et al. [83] introduced a multi-stage pipeline, which utilized stable diffusion and Low-Rank Adaptation for realistic synthetic surgical endoscopic image generation. This method was used to capture complex details and prevent the gap between real and generated data. Kim and Park [84] designed a Multiple Switchable Spatially Adaptive Normalization, denoted as MS-SPADE, which enhances and integrates into a latent Diffusion Model-based framework for 3D multimodal medical image-to-image translation, addressing the challenge of volumetric cross-modality synthesis. Mahdi et al. [85] provided a stable and high-accuracy 3D latent diffusion framework for MR-only radiotherapy, focusing on robust MRI-to-CT synthesis for clinically reliable synthetic CT generation. Kui et al. [86] proposed a novel medical latent variational Diffusion Model, denoted as Med-LVDM, designed for efficient medical image translation in multimodal and cross-modality settings. Specifically, a variational diffusion formulation was introduced based on weighted mean squared error to enable a more stable and interpretable optimization of the image translation process. Machacek et al. [87] proposed a mask-conditional latent Diffusion Model for gastrointestinal (GI) polyp image synthesis, where the generation process is guided by segmentation masks. Applying LDM-based methods to medical image generation has been extensively explored to enhance data diversity and improve model generalization. Existing studies suggest that latent Diffusion Models can learn complex anatomical representations and generate realistic volumetric medical images that preserve structural fidelity, which can strengthen downstream learning tasks when such synthetic data are integrated into training pipelines.

#### 3.5.2. Multimodal Generative AI: Text-to-Medical Image

The application of text-to-image synthesis is an emerging research direction in medical imaging. Recent research on the advancement of diffusion-based generative techniques such as Stable Diffusion and text-guided latent diffusion frameworks has led to increased interest in text-to-image synthesis. Text-conditional MR image generation was investigated by Kim et al. [88] using magnetic resonance images, enabling the generation of images directly from medical text descriptions. Furthermore, cross-attention map analysis was performed to interpret textual conditions and generated image features. Xu et al. [89] designed a hierarchical text-guided diffusion framework, denoted as MedSyn, which enhances low-resolution text-conditioned synthesis into a high-resolution 3D lung CT image generation system, while Khader et al. [90] applied the DDPM method for the synthesis of 3D CT/MRI medical images. Huang et al. [91] designed a lightweight transformer-based diffusion framework, denoted as Chest-Diffusion, that transforms report-guided text representations into a text-to-chest X-ray generation model for authentic and computationally efficient medical image synthesis, improving the quality of generated images. Kidder et al. [9] employed DreamBooth with improved text-to-image prompts for the generation of MRI and mammography images. In another study, Li et al. [92] applied controllable Diffusion Models to improve generative data augmentation in medical imaging, integrating lesion masks and text descriptions to generate anatomically consistent and semantically diverse synthetic images. Anaya et al. [93] highlighted the importance of prompt-conditioned Diffusion Models in medical image generation, showing that a Stable Diffusion framework trained on chest X-ray data and guided by ClinicalBERT embeddings achieves clinically meaningful image synthesis, while Xie et al. [94] fine-tuned Stable Diffusion Models, denoted as MedDiff-FT, designed to generate images using guided prompts. Montoya et al. [10] fine-tuned a pretrained Stable Diffusion Model for mammographic imaging, which generates quality images through text prompts and latent space processing.

#### 3.5.3. Privacy-Preserving Generative Models

Privacy regulations such as HIPAA and GDPR have strict restrictions on the acquisition, storage, and sharing of medical imaging data, which directly restrict access to large labeled datasets that are essential for training generative models used in clinical AI. Synthetic data generation aims to produce images that closely mimic real-world data distributions while ensuring privacy preservation. Several existing studies have explored privacy-preserving techniques in generative models for medical imaging, examining approaches such as differential privacy and federated learning [95,96,97]. The limitation of medical dataset availability was addressed by proposing “Privacy-Preserving Latent Diffusion-Based Synthetic Medical Image Generation”. A novel technique was introduced that integrates PF-VGG, a privacy filter, which removes generated images that are similar to real images while maintaining data privacy [98]. A differentially private WGAN-based method for synthetic radiology image generation was implemented, enabling the creation of high-quality and privacy-preserving medical images to support deep learning diagnosis tasks across distributed healthcare institutions without sharing or storing patient data on a central server [99]. Deepee extends differentially private deep learning to medical imaging applications for image classification and segmentation tasks while ensuring privacy. In Deepee, the training process is designed not only to learn accurate diagnostic representations but also to protect patient information [100].

## 4. Loss Functions in GANs

GANs are the most utilized generative approach in the reviewed studies. GAN loss function is important in guiding the training process for image synthesis. Several researchers have investigated GAN loss functions to enhance the visual quality. The choice of loss function in GANs impacts model training and performance, guiding the network in capturing relevant features, reducing instability, mode collapse, and non-convergence by stabilizing adversarial learning [101]. It becomes possible to achieve stable training and generate higher-quality images by improving GAN loss functions. The purpose of the vanilla GAN loss function is to minimize the difference between the generated and real images and is defined as in Equation (1):

(1) $$\min_{G}\max_{D}V\left(D,G\right)=E_{x∼p_{d}\left(x\right)}\left[\log D\left(x\right)\right]+E_{z∼p_{z}\left(z\right)}\left[\log\left(1-D\left(G\left(z\right)\right)\right)\right]$$

GANs [2] have several common failure modes, such as mode collapse and vanishing gradients caused by minimizing JSD between the generated and true data distributions. Nowozin et al. [102] replaced the standard GAN objective with formulations based on divergence to measure the difference between real and generated probability distributions. The divergence is defined in Equation (2):

(2) $$D_{f}\left(P∥Q\right)=\int_{X}q\left(x\right)\;f\;\left(\frac{p(x)}{q(x)}\right)\;dx$$

Mao et al. [103] replaced the standard GAN sigmoid cross-entropy loss with a least squares loss function for the discriminator to reduce the vanishing gradient problem, named LSGAN. It is defined in Equations (3) and (4):

(3) $$\begin{array}{cc} \min_{D}V_{\mathrm{LSGAN}}\left(D\right) & =\frac{1}{2}E_{x∼p_{\mathrm{data}}\left(x\right)}\left[{\left(D\left(x\right)-y_{\mathrm{real}}\right)}^{2}\right]\\ \; & \;+\frac{1}{2}E_{z∼p_{z}\left(z\right)}\left[{\left(D\left(G\left(z\right)\right)-y_{\mathrm{fake}}\right)}^{2}\right] \end{array}$$

(4) $$\min_{G}V_{\mathrm{LSGAN}}\left(G\right)=\frac{1}{2}E_{z∼p_{z}\left(z\right)}\left[{\left(D\left(G\left(z\right)\right)-y_{\mathrm{gen}}\right)}^{2}\right]$$

To address the problems of vanishing gradients and mode collapse, Arjovsky et al. [47] introduced Wasserstein GAN, which uses Wasserstein distance to assess the difference between synthetic and real probability distributions. Unlike JS-divergence and KL-divergence, which fail to provide meaningful gradients for disjoint distributions, earth mover distance maintains meaningful gradient information, as defined in Equation (5):

(5) $$W\left(P_{\mathrm{data}},P_{\mathrm{gen}}\right)=\underset{\gamma \in \Pi (P_{\mathrm{data}},P_{\mathrm{gen}})}{\inf}E_{(u,v)∼\gamma }\left[∥u-v∥\right]$$

Gulrajani et al. [104] introduced WGAN-GP, which used a different technique compared with the original WGAN. In WGAN, the authors faced Lipschitz constraint problems. To overcome this, WGAN-GP employs a gradient penalty in the loss function, optimizing the gradient penalty term, which is defined in Equation (6):

(6) $$L=E_{\tilde{x}∼P_{g}}\;\left[D(\tilde{x})\right]-E_{x∼P_{r}}\;\left[D(x)\right]+\lambda E_{\hat{x}∼P_{\hat{x}}}\left[{\left({∥\nabla_{\hat{x}}D\left(\hat{x}\right)∥}_{2}-1\right)}^{2}\right]$$

Table 6 provides the advantages and limitations of loss functions commonly used in research.

Qi et al. [105] introduced a loss-sensitive GAN by incorporating Lipschitz regularity to deal with the limitations of unstable training and poor generalization in standard GAN, and it is defined in Equation (7):

(7) $$\begin{array}{cc} \min_{D}V\left(D\right) & =E_{x∼P_{\mathrm{data}}\left(x\right)}\left[L_{\theta }\left(x\right)\right]\\ \; & \;+\lambda E_{x∼P_{\mathrm{data}}\left(x\right)}\left[\Delta \left(x,G\left(z\right)\right)+L_{\theta }\left(x\right)-L_{\theta }\left(G\left(z\right)\right)\right] \end{array}$$

Li et al. [106] designed a GAN-based framework by incorporating a hybrid loss function, including sharpness, perceptual, adversarial, and structural similarity loss for low-dose CT images, named SSWGAN. This study used WGAN for denoising and showed that SSWGAN achieved PSNR of 35.6 and SSIM of 0.95, and it is represented in Equation (8):

(8) $$\begin{array}{cc} L_{\mathrm{SSWGAN}} & =\alpha \;L_{\mathrm{WGAN}}\left(G,D\right)+\beta \;L_{\mathrm{Perceptual}}\left(G\right)\\ \; & \;+\gamma \;L_{\mathrm{Sharp}}\left(G\right)+\omega \;L_{\mathrm{SSIM}}\left(G\right) \end{array}$$

The proposed hybrid loss function help to preserve sharpness, texture, and anatomical detail and maintains structural similarity between normal and denoised CT images. Chen et al. [107] introduced an ICycle-GAN with an aim to maintain CT and MRI image sharpness in generated images for the segmentation task, incorporating dual generators, dual discriminators, and a correction network. They employed an encoder and decoder architecture as a correlation network to reduce feature blurring and preserve fine details. The ICycle-GAN loss function is defined in Equation (9):

(9) $$\begin{array}{cc} L_{\mathrm{GAN}}\left(G_{X\to Y},G_{Y\to X},D_{X},D_{Y}\right) & =L_{\mathrm{GAN}}\left(G_{X\to Y},D_{Y},X,Y\right)\\ \; & \;+L_{\mathrm{GAN}}\left(G_{Y\to X},D_{X},Y,X\right)\\ \; & \;+\lambda \;L_{\mathrm{cyc}}\left(G_{X\to Y},G_{Y\to X}\right) \end{array}$$

In CT → MRI stage, the loss $L_{GAN}\left(G,D_{Y},X,Y\right)$ evaluates the ability of the discriminator $D_{Y}$ to distinguish real MRI images and generated MRI images translated from the CT modality. Similarly, in MRI → CT stage, the loss $L_{GAN}\left(G,D_{X},Y,X\right)$ trains the discriminator $D_{X}$ to differentiate real CT images from the synthesized CT image generated from the MRI modality. Heng et al. [108] introduced HLSNC-GAN by combining adversarial loss, identity loss, cycle-consistency loss, and hinge loss function for CT and MRI image synthesis. Furthermore, switchable normalization and the RMSprop optimizer are employed to improve the quality and diversity of the image. It is formulated in Equation (10):

(10) $$\begin{array}{cc} L_{\mathrm{total}}\left(G,F,D_{1},D_{2}\right) & =L_{\mathrm{adv}}\left(G,D_{1}\right)+L_{\mathrm{adv}}\left(F,D_{2}\right)\\ \; & \;+\lambda \cdot L_{\mathrm{cycle}}\left(G,F\right)+\lambda \cdot L_{\mathrm{identity}}\left(G,F\right)\\ \; & \;+\lambda \cdot L_{D}+\lambda \cdot L_{G} \end{array}$$

Their proposed framework increased PSNR and SSIM, and eliminated inconsistencies between multiple modalities. Zheng et al. [109] introduced LFGAN for synthesizing CT liver tumors by reducing noise and distortion in images, while maintaining texture details. Moreover, GLCM is applied to measure correlation, contrast, and dissimilarity and employs reconstruction and hinge loss; it is formulated as stated in Equations (11) and (12):

(11) $$\begin{array}{cc} L_{\mathrm{GLCM}} & =\sum (\left|\mathrm{CON}(f)-\mathrm{CON}(x)\right|\\ \; & \;+\left|\mathrm{DIS}(f)-\mathrm{DIS}(x)\right|\\ \; & \;+\left|\mathrm{COR}(f)-\mathrm{COR}(x)\right|) \end{array}$$

(12) $$\begin{array}{cc} L_{D} & =-E_{x∼P_{\mathrm{real}}}\left[\min\left(0,-1+D(x)\right)\right]\\ \; & \;-E_{\hat{x}∼G\left(z\right)}\left[\min\left(0,-1-D(\hat{x})\right)\right]\\ \; & \;+L_{\mathrm{recons}}+L_{\mathrm{GLCM}} \end{array}$$

In addition, the authors compared classification models, including MobileNetV2, SqueezeNet, ResNet34, ConvNeXt, and ShuffleNetV2, to assess their effectiveness using accuracy, sensitivity, specificity, and AUC. Gajera et al. [110] introduced a GAN-based network by incorporating the Charbonnier loss for CT-scan denoising, named CL-GAN, and it is formulated as in Equation (13):

(13) $$L_{CL}=C\left(\hat{x},x\right)=\sum_{i}\sum_{j}\sqrt{{\left({\hat{x}}_{i,j}-x_{i,j}\right)}^{2}+\epsilon^{2}}$$

Furthermore, the proposed hybrid loss function combined WGAN loss, perceptual loss, and Charbonnier loss, and is formulated in Equation (14):

(14) $$\min_{G}\{\lambda_{1}\left[\max_{D}L_{\mathrm{WGAN}}\left(D,G\right)\right]+\lambda_{2}L_{\mathrm{PL}}\left(G\right)+\left(1-\lambda_{1}-\lambda_{2}\right)L_{\mathrm{CL}}\left(G\right)\}$$

The results demonstrate superior performance compared with baseline synthetic methods. Li and Jiang et al. [111] utilized SRGAN by combining Texture loss for super-resolution image synthesis, named TSRGAN. This texture loss used the Gram matrix, a statistical representation of feature correlations, that captures texture information. In addition, WGAN-GP was employed to enhance adversarial training. They combined perceptual loss, content loss, and texture loss to refine image quality, and it is formulated in Equation (15):

(15) $$L_{G}=\lambda_{\mathrm{per}}L_{\mathrm{per}}+\lambda_{\mathrm{tex}}L_{\mathrm{tex}}+\lambda_{\mathrm{adv}}L_{\mathrm{adv}}+\lambda_{\mathrm{con}}L_{\mathrm{con}}$$

According to their experiments, the TSRGAN method achieved promising results, with an average PSNR of 27.99 dB and an SSIM of 0.77. Similarly, Zhao et al. [112] combined a denoising CNN with SRGAN for super-resolution image reconstruction. By incorporating MSE loss, VGG loss, and WGAN loss, they achieved high-quality images while reducing noise in the generated outputs, and the total loss is formulated in Equation (16):

(16) $$L_{SR}=L_{SR}^{C\text{-}\mathrm{MSE}}+\lambda_{1}L_{SR}^{\mathrm{VGG}}+\lambda_{2}L_{SR}^{\mathrm{WGAN}}$$

The proposed texture loss is formulated in Equation (17):

(17) $$L_{\mathrm{tex}}={∥G\;\left(\phi \;\left(I^{\mathrm{gen}}\right)\right)-G\;\left(\phi \;\left(I^{\mathrm{HR}}\right)\right)∥}_{2}^{2}$$

Performance metrics, including SSIM and PSNR, were employed, yielding an SSIM of 0.9 and a PSNR of 30.4. Wang et al. [113] utilized an encoder–decoder architecture as a generator network to enhance image quality in a low-light environment and designed a multiscale feature extraction method to collect more informative details. The multiscale feature module comprises a convolution layer, max, and average pooling. In addition, a dual-discriminator is introduced comprising local and global discriminators to improve generator performance. By incorporating perceptual and color loss in the generator, the visual quality, perceptual similarity, and color correctness of the images are enhanced. It is formulated in Equation (18):

(18) $$\mathrm{Loss}=L_{\mathrm{GAN}}+L_{\mathrm{per}}+L_{\mathrm{color}}$$

Akhmedova and Korber [29] presented GANetic loss for improving GAN training using Genetic programming, which are employed as an optimization problem. The loss function in their approach consists of Wasserstein, Least Squares, Binary Cross-Entropy, and Hinge loss, improving image generation and anomaly detection tasks. The proposed loss is formulated in Equation (19):

(19) $$L_{\mathrm{GANetic}}=\frac{1}{N}\sum_{i=1}^{N}\left({\left(y_{\mathrm{pred}}^{\left(i\right)}\right)}^{3}+\sqrt{\frac{\alpha \;y_{\mathrm{real}}^{\left(i\right)}}{y_{\mathrm{pred}}^{\left(i\right)}+\epsilon }+\epsilon }\right)$$

The model was trained on the CIFAR-10 and MNIST datasets using the DCGAN architecture. Furthermore, it is validated on Hyper-Kvasir, BreCaHAD, and LAG for image synthesis. Xiao et al. [114] presented a Monte Carlo GAN (MCGAN) algorithm to improve generator training. Regression loss is introduced to minimize MSE between real and generated samples. The performance of MCGAN is evaluated on diverse data types, including image generation, video generation, and time series data, and demonstrates superior performance in both conditional and unconditional training. It is formulated in Equation (20):

(20) $$L_{R}\left(\theta ,\phi \right)=E_{(a,b)∼\mu }{\left[D_{\phi }\left(a\right)-E_{\hat{a}∼\nu_{\theta }\left(b\right)}\left[D_{\phi }\left(\hat{a}\right)\right]\right]}^{2}$$

where μ denotes the true data distribution, $v_{\theta }$ represents the generated data distribution and $D_{\phi }$ is the discriminator. Guo et al. [115] designed an adaptive GAN to address the complexity in generating RGB images and employed Wasserstein loss to measure convergence, named MedGAN. Their findings show that the MedGAN model, which is trained adaptively, achieves better visual quality and faster training of generated images, outperforming WGAN. Gonzalez et al. [116] designed an evolutionary technique by identifying optimal parameters to improve the quality of generated images and mode collapse in GANs, named TaylorGAN. Jin et al. [117] designed a model for accurate and high-visual-quality image synthesis, named HMS-MambaGAN. The author initially trained HMS-MambaGAN using multimodal datasets and simultaneously employed a novel Gray-Level Gradient Co-occurrence Matrix (GLGCM)-based loss function to enhance the textures and realism of the images. Experimental results demonstrate better performance compared with existing medical image synthesis models and are formulated in Equation (21):

(21) $$\begin{array}{cc} L_{G_{1}} & =L_{\mathrm{GAN}}+\lambda_{1}L_{L1}+L_{\mathrm{HisGDL}}+L_{\mathrm{GLGCM}},\\ L_{G_{2}} & =L_{\mathrm{GAN}}+\lambda_{1}L_{L1}+L_{\mathrm{HisGDL}},\\ L_{G} & =L_{G_{1}}+L_{G_{2}}. \end{array}$$

Table 7 provides a review of loss functions employed in GAN-based image generation.

Goceri [118] employed a conditional GAN to augment dermoscopy images for classification tasks. A hybrid loss function, including content loss, L1 loss, and structural similarity loss, was introduced to improve the generator’s ability, improving classification accuracy to 93.12%. Veiner et al. [119] introduced a unifying α-parametrized generator loss function to address the unstable training in GANs. This model employed a canonical discriminator loss and a generator loss based on a function $L_{\alpha }$. Under an optimal discriminator, the generator’s objective reduces to minimizing a Jensen–$f_{\alpha }$ divergence, which generalizes the Jensen–Shannon divergence.

## 5. Discussion

This review examines the recent developments in medical image generation using generative models. The generative frameworks, including Diffusion Models, GANs, and VAEs, various GAN loss functions, medical image modalities, and datasets were examined.

### 5.1. Generative Imaging Modalities

Generative models (VAE, GAN, and Diffusion Models) have been applied to various imaging modalities and are capable of producing high-resolution, realistic images for classification, diagnosis, and segmentation [6,26,30,38,43]. In our analysis, most studies applied GANs to enhance and generate diverse medical datasets. Additionally, generative models have been applied across multiple imaging modalities to enhance low-dose CT and MRI scans, generate realistic chest X-rays, and improve endoscopic and dermoscopic images, providing a consistent framework for evaluation and comparison [27,32,67,68].

A large number of studies have been published in the domain of gastrointestinal endoscopy, focusing on high-quality image synthesis, lesion detection, and classification. Endoscopy procedures provide high-resolution visualization of mucosal surfaces, lesions, and polyps for detection. Using multi-model endoscopy imaging, assessment is performed by integrating information from multiple imaging sources, which can introduce systematic errors due to differences in acquisition, lighting, and imaging angle. Several researchers have employed generative models, including cGAN, StyleGAN, Pix2Pix, BlobGAN, VAE-GAN, and Diffusion Models for endoscopy image synthesis [12,24,25,26,27,28,29].

MRI and CT are used in radiotherapy for treatment planning. MRI is a widely used imaging modality applied in brain, cancer, pelvis, and soft tissue imaging, and existing techniques fail to restore fine details. Numerous researchers have investigated MRI image synthesis to achieve high-quality MRI scans for better diagnosis [8,32,33,34,77]. Generative models can produce diverse MRI images, improving image quality, reducing noise, and enhancing resolution. Various generative models have been employed for brain MRI synthesis; for example, Feng et al. [39] employed a deep CNN as a generator, while Dorent et al. introduced a multimodal hierarchical VAE. Various CycleGAN-based imaging techniques have been proposed for transforming CT images into MRI and other imaging modalities, such as ultrasound, Positron Emission Tomography (PET), Optical Coherence Tomography (OCT), and cervical imaging, where images are synthesized by learning the cross-modality [5,67].

The findings suggest that generative models, particularly GAN-based methods, have higher fidelity and realism than Diffusion Models and Variational Autoencoders on the same diverse medical imaging modalities. They found that generative-based methods were more accurate in improving low-dose and noisy scans and enabled realistic augmentation of chest X-rays, MRI, CT, and endoscopy images than diffusion-based methods, while requiring fewer computational resources and time.

### 5.2. Comparative Analysis

GANs and Diffusion Models provide complementary advantages in order to achieve high-quality and realistic images. GANs provide high-quality, realistic, and sharp image details due to the discriminator–generator training process, while Diffusion Models focus on the iterative denoising process, and the generated results improve structural consistency and finer detail preservation. VAEs provide a structured probabilistic framework with a continuous latent space but produce lower image sharpness. Numerous researchers have applied generative models for image generation tasks, for instance, Refs. [67,68,69] have applied Variational Autoencoders to multiple medical imaging modalities, such as lung cancer, cardiac MRI, and lumbar spine image synthesis.

From a speed perspective, the authors of [120] compared GANs and Diffusion Models within a similar training setup. They generated 100,000 images for model training with the diffusion-based method and the GAN-based method. The Diffusion Model required approximately 1.5 days, whereas the GAN required approximately 10 min. From a stability perspective, GANs are sensitive to training dynamics due to their adversarial networks; although Diffusion Models provide improved stability, they involve more complex architectures and require longer processing times compared with GAN-based approaches. In contrast, VAEs tend to exhibit stable training, indicating a trade-off in image sharpness. Table 8 summarizes the evaluations of image quality, training stability, computational cost, output diversity, and controllability.

Diffusion-based models rely on an incremental denoising process that preserves structural similarity and texture details from Gaussian noise; however, they are computationally intensive and exhibit slower inference times. GAN-based architectures focus on one-shot generation of high-quality images. By employing an adversarial training process, GANs can generate realistic, high-quality, and sharp images; however, they fail to capture data diversity due to mode collapse and are sensitive to hyperparameters and network design. Within a domain of generative models for medical image synthesis, GANs stand out for their ability to generate sharp and visually realistic images, but Diffusion Models generally achieve higher image fidelity and improved structural consistency. In contrast, VAEs produce blurrier outputs and can struggle with capturing high variability in complex datasets. Lastly, in terms of computational cost, VAEs and GANs generally require moderate resources due to their architectural and training requirements, while Diffusion Models are computationally very expensive and require substantial resources. GANs also maintain realistic anatomical characteristics and fine-grained details but introduce training instability and mode collapse. First, the GAN discriminator provides pixel-level feedback, which is essential for accurately representing mucosal surfaces. BlobGAN with self-attention achieved FID 15.5 for gastrointestinal image generation [25], and SCAGAN achieved FID 31.349 for wireless capsule endoscopy [5], representing the lowest FID scores across all models for the gastrointestinal imaging task in the reviewed literature. GANs showcase high computational efficiency capabilities, generating high-quality realistic images with low computational cost [120], which is key for large-scale data augmentation in resource-constrained settings. GANs can generate fewer synthetic tumor images for brain MRI, leading to reduced downstream performance, which requires more diverse and accurate data synthesis. Diffusion Models outperform GANs in SSIM. Ma et al. [8] used cross-attention and a multi-level U-Net for Brain MRI image synthesis, achieving an SSIM of 0.915, while Feng et al. [39] used knowledge distillation GANs for Brain MRI image synthesis and achieved an SSIM of 0.593. In contrast with GANs, Diffusion Models generate complex and rare anatomical images using an iterative denoising process. For cervical spine MRI synthesis [34], it learns complex patterns and integrates Gaussian–point noise, which improves inter-vertebral disc boundary preservation, while GANs may not fully capture these structures due to mode collapse. Stable Diffusion has also been widely used, and when fine-tuned with ControlNet, it produces clinically viable high-resolution ultrasound images. Table 9 presents advantages and limitations of generative models.

The quantitative synthesis of FID, SSIM, and PSNR [121] values presented in Table 10 shows clear performance across generative model categories and target organs.

### 5.3. Limitations of GAN in Image Generation

Numerous researchers have implemented various approaches in order to address the challenges associated with GAN training, including non-convergence, vanishing gradient, mode collapse, and training instability.

#### 5.3.1. Model Collapse

Mode collapse is one of the most important problems in GAN training. Mode collapse occurs in GANs when the generator produces the same type of images and fails to capture the diversity of the training data, while the discriminator can still distinguish between real and generated samples. As a result, improving the diversity of synthetic medical images remains a challenging task for researchers. This is a common issue in GAN-based medical image generation. This limitation reduces GAN use in applications such as MRI, CT, or endoscopy image synthesis. Several researchers have proposed loss functions and training strategies in order to reduce mode collapse [47,123]. Despite these efforts, mode collapse still occurs in many GAN-based medical image generation models.

#### 5.3.2. Vanishing Gradient

A significant challenge in GAN training is that the generator receives a small gradient signal and fails to update its parameters when the discriminator is too strong. The generator updates parameters based on discriminator feedback during training. As a result, achieving stable and effective training of GANs remains a challenging task for researchers. This limitation reduces image quality, diversity, and impact on clinical applications. In order to prevent vanishing gradient, researchers have designed various methods, including loss function, gradient penalty, and normalization techniques [47,103,104].

#### 5.3.3. Training Instability

GANs suffer from training instability due to adversarial training. When the generator and discriminator fail to converge, training instability arises. Additionally, hyperparameter selection, with its impact on learning rates, batch sizes, and optimizer settings, is critical when stable and effective GAN training is required. As a result, maintaining diversity in the generated images remains a significant challenge for researchers. Researchers have applied spectral normalization, gradient penalty, and other regularization techniques to improve the quality of the generated image. In order to overcome instability and convergence issues, Arjovsky et al. [47] have proposed the Wasserstein GAN, and Mao et al. [103] introduced the Least Squares GAN. Miyato et al. [124] introduced a stabilizing framework with a spectral normalization technique that constrains the Lipschitz constant of the discriminator. In a related study, Zhao et al. [125] introduced a Differentiable Augmentation technique to address the instability due to data scarcity. The Differentiable Augmentation model applied a consistent set of differentiable transformations, including translation, color jittering, and cutout, to both real and generated samples. This approach delivered more stable convergence and improved generator performance than previously employed augmentation strategies.

### 5.4. Addressing the Research Questions

This systematic review addresses the four formulated research questions as follows:

- RQ1 (Generative Techniques vs. Modalities): This question is answered in Section 3 (Medical Image Synthesis with Generative Models). We have categorized all three generative architectures, such as GAN, VAE, and Diffusion Models, across MRI, CT, X-ray, endoscopic modalities, and other medical image modalities. A comprehensive breakdown of these techniques, including specific author contributions from 2021–2026.
- RQ2 (Loss Functions in GANs): The specific objective functions, adversarial losses, and hybrid loss formulations used to stabilize training and improve structural similarity or pixel-level accuracy are detailed in Section 4 (Loss Functions in GANs).
- RQ3 (Clinical Applications): The translation of these models into clinical settings such as gastrointestinal lesion detection, gastric cancer synthesis, and multimodal diagnostics is explored throughout the subsections of Section 3. Lee et al. [45] deployed StyleGAN3, designed to synthesize gastric cancer images and further integrated with YOLOv8 detection, achieving 93.98% sensitivity in clinical-grade evaluation. Yoon et al. [44] applied GAN-generated colonoscopy images to improve detection performance. Wang et al. [82] proposed MINIM, a unified text-to-medical-image framework evaluated across OCT, fundus, chest CT, and chest X-ray in clinical imaging contexts. Shi et al. [98] designed a privacy-preserving filter with the aim of addressing clinical deployment constraints in real-world medical settings. Diffusion-based augmentation exhibits high-quality image generation performance for mammography (Montoya et al. [10]) and lung CT (Krishna et al. [31]), demonstrating its applicability to diagnostic decision support. Despite growing adoption of medical image synthesis approaches, a fundamental gap remains between automated image quality metrics and clinically meaningful radiological assessment, and their ability to match real clinical evaluation standards is limited. This is an important limitation, as commonly used evaluation metrics such as FID, SSIM, and PSNR measure statistical or pixel-level similarity between generated and real images rather than true clinical diagnostic value. Lee et al. [45] validated a gastric cancer generated images using YOLOv8 detection pipeline. The results demonstrate that StyleGAN3-generated images achieve 93.98% sensitivity for downstream clinical task performance. Similarly, Yoon et al. [44] validated GAN-generated colonoscopy images through detection performance rather than radiologist scoring. Wang et al. [82] evaluated MINIM across four clinical imaging modalities using AUROC as a downstream clinical metric, which is closer to diagnostic utility than FID alone.
- RQ4 (Overcoming Data Limitations): The methodologies for utilizing synthetic data are addressed in Section 2 (Datasets Overview, Table 4). GAN-based methods are used to generate synthetic images from small and imbalanced training datasets (Saimon et al. [53], Ding et al. [126], Feng et al. [39]). Transfer learning and fine-tuning of pretrained models on small medical datasets was employed in diffusion-based studies, including DreamBooth fine-tuning (Kidder et al. [9]) and Stable Diffusion fine-tuning (Montoya et al. [10]). Multimodal image synthesis was used to generate missing imaging modalities from available datasets, reducing the need for acquiring additional data (Wang et al. [26], Afnan et al. [79]).

## 6. Limitations

Despite providing a comprehensive comparative synthesis of GANs, Diffusion Models, Variational Autoencoders, and Hybrid architectures for synthetic medical image generation, this review still has several limitations. The review protocol was not pre-registered in PROSPERO or a similar public registry, which may introduce a minor risk of bias. The included studies are representative but demonstrate relatively limited diversity in dataset size and imaging modality. In addition, most included studies only reported clinical findings based on a single automated image quality metric (eg, FID, SSIM, PSNR) compared with evaluations performed by radiologists, without further robust downstream clinical validation, which limits the strength of the conclusions. These limitations should be considered when interpreting the synthesized findings.

## 7. Conclusions

This SLR provides a comprehensive overview of Generative AI models for medical image generation and further examines GAN loss functions for producing high-quality images. Researchers have incorporated these models for synthesizing rare medical images, the complexity of medical structures and the scarcity of data are still major challenges. While the Diffusion Models can generate high-quality images, they are computationally very expensive, and VAEs face challenges in generating complex medical images. However, GANs offer faster image generation and lower computational requirements while maintaining high visual quality. This review provides an in-depth analysis of various generative models and GAN loss functions commonly used in the domain of medical image generation.

## Figures and Tables

**Table 1:** PICOC Search Strategy.

PICOC Elements	Keywords
Population (P)	“medical imaging”, “clinical imaging”
Intervention (I)	“image generation”, “image synthesis”, “synthetic image”, “GAN”, “variational autoencoder”, “diffusion model”, “DDPM", “stable diffusion”, “latent diffusion models”
Comparison (C)	“Diffusion Model”, “Generative Adversarial Network”, “Variational Autoencoders”
Outcome (O)	“image quality”, “FID score”, “SSIM”, “PSNR”
Context (C)	“medical image synthesis”, “healthcare AI”, “clinical diagnosis”, “disease detection”, “rare disease imaging”

**Table 2:** Inclusion and Exclusion Criteria for Study Selection.

Criteria Type	Description
Inclusion Criteria (IC)	IC1: Peer-reviewed full-text journal articles and conference proceedings. IC2: Studies focus on medical image generation or synthesis using GANs, Diffusion Models, or Autoencoders. IC3: Must be in English. IC4: Papers published between 2021 and 2026
Exclusion Criteria (EC)	EC1: Preprints (e.g., non-peer-reviewed arXiv papers), abstracts, short papers. EC2: Studies not focusing on clinical or medical imaging data generation tasks. EC3: Manuscripts with full text unavailable or not written in English. EC4: Literature published prior to 1 January 2021.

**Table 3:** Generative Models and Variants for Medical Imaging.

Model Type	Variants
GAN-based architecture	Deep Convolutional GAN, Denoising GANs, StyleGAN, Cycle GAN, Pix2Pix, conditional GAN, PGAN, super-resolution GAN
Diffusion Models	Stable Diffusion, Denoising Diffusion Probabilistic Model (DDPM), Latent Diffusion Model (LDM)
Autoencoder	Convolutional VAE (CVAE), Conditional VAE, VAE

**Table 4:** Overview of Datasets, Total Images, and Domain.

Dataset	Domain	Total Images	Year Released	References
Kvasir-v2	Gastrointestinal (Endoscopy)	8000	2017	[12,24,25,26]
KID	Gastrointestinal (Capsule Endoscopy)	2371	2017	[27,28]
Kvasir-Capsule	Gastrointestinal (Capsule Endoscopy)	47,238	2020	[5,28]
Hyper-Kvasir	Gastrointestinal (Endoscopy)	110,079	2020	[29]
Kvasir-SEG	Polyp Segmentation	1000	2020	
MedicalMNIST	Multi-domain	58,954	2017	[30]
NLST (National Lung Screening Trial)	Lung-CT	50,000	2014	[31]
BraTS	MRI	369 cases	2020	[32,33]
Cervical Spine MRI Dataset	MRI (Cervical Spine)	514	2022	[34]
Knee Osteoarthritis	Knee X-ray	5778	2018	[30]
TotalSegmentator	CT	1204	2023	[35]

**Table 5:** Quality Assessment (QA) Criteria for Literature Selection.

QA ID	Quality Assessment Question	Scoring Criteria
QA 1	Is the proposed generative architecture and objective clearly described?	1: Objectives clearly defined.
		0.5: Partially specified.
		0: Not specified.
QA 2	Is the methodology (architecture, loss functions, datasets) defined clearly?	1: Methodology is defined.
		0.5: Partially elaborated.
		0: Not elaborated.
QA 3	Does this research focus on medical image generation?	1: Focuses on medical images.
		0.5: Partially elaborated.
		0: Non-medical.
QA 4	Are the results evaluated using standard metrics (FID, SSIM, PSNR)?	1: Results fully evaluated.
		0.5: Partially discussed.
		0: Not evaluated.
QA 5	Are the limitations and findings clearly concluded?	1: Concluded clearly.
		0.5: Partially concluded.
		0: No conclusion.

**Table 6:** Pros and Cons of Commonly Used GAN Loss Functions.

Loss Function	Pros	Cons
Minimax GAN	Produce quality synthetic images; Easy to implement	Mode Collapse; Training Instability; Vanishing Gradient
WGAN	Improve training stability and reduced mode collapse	Weight Clipping; Computational Complexity; Sensitive Hyperparameters
WGAN-GP	Generates higher-fidelity images than WGAN: Improves training stability and convergence	Slow Convergence; Longer Processing Time
LSGAN	Generates quality images; Faster Convergence	Limited output diversity
EBGAN	Prevents mode collapse; Stabilizes training	Lower image quality: Autoencoder-dependent performance

**Table 7:** Summary of Loss Functions.

Category	Purpose	Loss Functions
Adversarial Loss [2,108,109]	Improves the realism of generated samples through adversarial training	Vanilla GAN, WGAN, WGAN-GP, LSGAN, Hinge Loss
Perceptual Loss [106,110,111,112,113]	Preserves high-level semantic features and visual textures	VGG Perceptual Loss, Perceptual Loss
Structural Loss [106,107,108]	Maintains anatomical structures and spatial relationships	SSIM Loss, Cycle Consistency Loss
Color Loss [108,113]	Ensures accurate color representation in generated images	Color Loss, Identity Loss
Textural Loss [109,111,117]	Captures fine-grained textural features in generated images	GLCM Loss, GLGCM Loss, Texture Loss
Pixel-Level Loss [110,111,112]	Minimizes pixel-level differences between images	L1 Loss, L2 Loss, Charbonnier Loss
Edge Loss [106,117]	Preserves image boundaries and fine details	Sharpness Loss, Gradient Loss
Content Loss [111,112]	Preserves important content-related features	L1 Loss, L2 Loss

**Table 8:** Comparison of GANs, Diffusion Models and VAEs.

Comparison Criterion	GANs	Diffusion Models	VAEs
Image Realism	Produces high-fidelity images with enhanced realism through adversarial learning.	Achieves higher overall image quality and better preserves global anatomical structure.	VAEs produce quality images, but image sharpness can be affected due to pixel-wise reconstruction loss.
Training Stability	Unstable during training compared with VAEs, and can suffer from issues such as mode collapse and sensitivity to hyperparameters.	More stable than GANs during training, but requires many denoising steps for image generation.	Generally stable with good convergence behavior but may suffer from posterior collapse due to the loss formulation.
Computational Cost	Training GANs requires moderate computational cost due to adversarial learning.	High computational cost due to fundamental constraints in their multi-step sampling process required for image generation.	Requires moderate computational cost due to the encoder–decoder architecture and sampling from a latent space.
Output Diversity	Produces diverse and high-quality images but may suffer from mode collapse, leading to reduced diversity.	Produces diverse and high-quality images with strong representation of varied data features.	Generate diverse images but may struggle with capturing high diversity and rare patterns in complex datasets.

**Table 9:** Pros and Cons of Generative Models.

Architecture	Pros	Cons
GAN Adversarial generator-discriminator training	Produces sharp, high-quality, and realistic images; fast generation	Mode collapse and limited diversity; Sensitive to hyperparameters; training instability; Vanishing Gradients
Diffusion Models Iterative denoising forward and reverse process	Preserves structural and semantic details; captures diverse modes; stable training	High computational cost; complex architecture; longer training time; Slow inference
Variational Autoencoders Encoder–Decoder architecture	Stable training; structured latent space	Produce blurry images, risk of posterior collapse, and limited fine-grained detail generation.

**Table 10:** Reported FID, SSIM, PSNR, and KID Values Across Imaging Modalities and Generative Models.

Modality	Model	Metric(s) Reported	Refs.
**MRI**			
Brain MRI	GAN	SSIM = 0.593	[39]
Brain MRI	Diffusion	SSIM = 0.915, PSNR = 36.16	[8]
Knee MRI	Diffusion	SSIM = 0.899, PSNR = 36.24	[8]
Alzheimer’s MRI	Diffusion	SSIM = 0.888, PSNR = 31.03	[8]
Cardiac MR (SR)	GAN	SSIM = 0.90, PSNR = 30.4	[112]
**CT Imaging**			
Abdominal CT	Diffusion	SSIM = 0.576, PSNR = 19.51	[35]
Low-dose CT	GAN	SSIM = 0.85, PSNR = 28.89	[106]
**Endoscopy/GI**			
Endoscopy (Kvasir)	GAN	SSIM = 0.83, PSNR = 31.86	[26]
Endoscopic	Diffusion	FID = 79.80	[83]
Endoscopy/GI	GAN	FID = 15.552	[25]
Capsule Endoscopy	GAN	FID = 31.349	[5]
Colonoscopy	GAN	FID = 42.19	[44]
WCE Capsule	VAE	FID = 81.9	[27]
**Ultrasound**			
Ultrasound	VAE	FID = 55.95	[122]
**Wireless Capsule Endoscopy**			
WCE Capsule	VAE	KID = 89.4	[28]

## Data Availability

This systematic review did not generate or analyze any new datasets.

## Supplementary Materials

The following supporting information can be downloaded at: https://www.mdpi.com/article/10.3390/diagnostics16142166/s1, Table S1: Full study characteristics and generative models grouped by application domain. File S1: PRISMA 2020 checklist.

## Abbreviations

The following abbreviations are used in this manuscript:

GAN	Generative Adversarial Network
VAE	Variational Autoencoder
DDPM	Denoising Diffusion Probabilistic Model
LDM	Latent Diffusion Model
FID	Fréchet Inception Distance
SSIM	Structural Similarity Index Measure
PSNR	Peak Signal-to-Noise Ratio
MRI	Magnetic Resonance Imaging
CT	Computed Tomography
PET	Positron Emission Tomography
OCT	Optical Coherence Tomography
WCE	Wireless Capsule Endoscopy
SLR	Systematic Literature Review
PRISMA	Preferred Reporting Items for Systematic Reviews and Meta-Analyses
WGAN	Wasserstein GAN
LSGAN	Least Squares GAN
CNN	Convolutional Neural Network
SRGAN	Super-Resolution GAN
